# A novel cohort of cancer-testis biomarker genes revealed through meta-analysis of clinical data sets

**DOI:** 10.18632/oncoscience.37

**Published:** 2014-05-06

**Authors:** Stephen J. Sammut, Julia Feichtinger, Nicholas Stuart, Jane A. Wakeman, Lee Larcombe, Ramsay J. McFarlane

**Affiliations:** ^1^ School of Medical Sciences, Bangor University, Bangor, UK; ^2^ Institute for Knowledge Discovery, Graz University of Technology, Austria; ^3^ Core Facility Bioinformatics, Austrian Centre of Industrial Biotechnology, Austria; ^4^ North West Cancer Research Institute, Bangor University, Bangor, UK; ^5^ NISCHR Cancer Genetics Biomedical Research Unit

**Keywords:** Cancer/testis antigens, cancer biomarkers, meiCT, gene expression, oncogenes, meiosis

## Abstract

The identification of cancer-specific biomolecules is of fundamental importance to the development of diagnostic and/or prognostic markers, which may also serve as therapeutic targets. Some antigenic proteins are only normally present in male gametogenic tissues in the testis and not in normal somatic cells. When these proteins are aberrantly produced in cancer they are referred to as cancer/testis (CT) antigens (CTAs). Some CTA genes have been proven to encode immunogenic proteins that have been used as successful immunotherapy targets for various forms of cancer and have been implicated as drug targets. Here, a targeted *in silico* analysis of cancer expressed sequence tag (EST) data sets resulted in the identification of a significant number of novel CT genes. The expression profiles of these genes were validated in a range of normal and cancerous cell types. Subsequent meta-analysis of gene expression microarray data sets demonstrates that these genes are clinically relevant as cancer-specific biomarkers, which could pave the way for the discovery of new therapies and/or diagnostic/prognostic monitoring technologies.

## INTRODUCTION

Achieving effective treatments for cancers is more difficult once the disease has reached the metastatic stage. This, in combination with the trend towards personalised approaches to cancer medicine means there is an increasing need to identify and develop cancer-specific biomarkers that can be employed in the development of early, pre-metastatic diagnostic and treatment strategies [1-7]. Additionally, advances in tumour immunology have reignited interest in cancer immunotherapeutics, such as cancer vaccines, adoptive therapeutics and targeted drug delivery using antibody-drug conjugates [8-17].

Cancer/testis antigens (CTAs) have emerged as a group of proteins that have significant immunogenic and cancer-specific potential [18-27]. *Bona fide* CTA genes are defined by having expression restricted to the testes in normal adult males, but are also aberrantly activated in cancers of either gender [18-27]. CTA genes are of importance for two fundamentally distinct reasons. Firstly, there is an immunological barrier, known as the blood-testis barrier, which generates an immunological privilege within the testis that is enforced via a number of pathways [28,29]; consequently, testis antigens that normally reside in an immunologically privileged setting are capable of eliciting an autologous immune response in the peripheral blood/tissues. Thus, CTAs can serve as immunologically restricted cancer-specific antigens, making them exceptionally attractive as diagnostic, prognostic and therapeutic biomarkers/targets, the targeting of which should not induce deleterious side effects to non-cancerous somatic tissue. For example, the activation of a specific cohort of such genes has been correlated with more aggressive lung cancers [30]. Additionally, use of an immunohistochemical approach in non-small cell lung cancers revealed a correlation between survival and the presence of known CTAs [31]. Of note, the presence of the CTA NY-ESO-1 correlated with an increased response to and benefit from neoadjuvant, and adjuvant chemotherapy respectively [31]. NY-ESO-1 is one of the most immunogenic CTAs and has been used as a successful target for adoptive therapy in the treatment of malignant melanoma [32].

Secondly, genes whose products normally serve to drive meiotic chromosome dynamics, germ cell regulation and gametogenic differentiation may have powerful oncogenic transforming activity if aberrantly expressed in non-germline, somatic tissues; a possibility that remains largely unexplored. The aberrant activation of these genes may confer biological processes that are advantageous to cancer cells but at the same time may be open to exploitation by therapeutic targeting (for examples, see 33,34). Indeed, increasing evidence indicates that a soma-to-germline transition could be a potential broad-spectrum oncogenic driver [35-39].

CTA genes have been classified based on expression profiles in normal tissues. These groups are: testis-restricted, testis-selective, testis/brain [or central nervous system (CNS) tissue]-restricted and testis/brain (CNS tissue) selective respectively [40]. Most of the known CTAs are encoded for by genes on the X chromosome (X-CT genes) [18,26,40], and many belong to large paralogous gene families (for example, see 41). Previously, meiosis-specific genes have been identified as CTA genes (for example, see 42,43), but recently a more systematic study described an extensive set of putative meiosis-specific genes, meiCT (or meiCTA) genes, as cancer/testis (CT) genes [44]; many of these are autosomally encoded, fitting with the fact that the X chromosome becomes transcriptionally silenced in mammalian male meiosis [45,46].

Given the strict cancer specificity of CTAs, the identification of new CT genes has exceptional therapeutic and biomarker potential. In this study, we describe a novel sub-category of meiCT genes, which have clinical importance, as demonstrated through meta-analysis of a clinically-relevant gene expression microarray data sets.

## RESULTS

A bioinformatics pipeline was previously established to identify putative human meiosis-specific genes that could potentially encode CTAs. This was based initially on a cohort of mouse genes predicted to be associated specifically with meiosis and spermatocyte development [44]. High stringency human orthologue identification and filtering for mitotic expression resulted in 375 human genes which were potentially testis spermatocyte /meiosis-specific [44]. These genes were then evaluated using an EST analysis pipeline based on the complete Unigene database [44]. Briefly, if a candidate gene was represented in a non-testis/non-central nervous system (CNS) normal tissue EST library, then it was excluded. The remaining genes were assessed further to see if they were represented in cancer EST libraries. From the original 375 potential testis-specific genes the EST analysis identified 177 candidate genes, of which 9 were testis-restricted, but also gave a positive cancer EST signature (class 1); 75 were testis-restricted, with no cancer EST signature (class 2); 21 were testis/CNS-restricted, with a positive EST signature (class 3) and 72 were testis/CNS-restricted, with no cancer EST signature (class 4).

We have previously defined the meiCT genes based on validation and gene expression microarray analyses of the class 1-3 genes [44]. Within the initial class 1-3 predicted gene sets RT-PCR validation revealed that a number were actually expressed in extensive somatic tissues [44]. Given this, we re-analysed our predicted class 4 genes using an updated CancerEST pipeline [47] and from this we identified 54 putative class 4 genes, those with expression signatures only in the testis and CNS of healthy tissue (Supplementary Table).

The gene expression profiles of the 54 candidate genes were validated using RT-PCR, initially on RNA isolated from a range of normal human tissues obtained *post mortem*, including testicular RNA. Of the 54 genes, 21 were expressed in more than two non-testis/non-CNS normal tissues and were therefore dismissed at this stage. Of the remaining 33 genes (bold in Supplementary Table), 30 had expression limited to the testis in normal tissue, 2 had expression limited to the testis and normal CNS tissues and 1 further gene had expression in one or two normal tissues in addition to testis, with or without CNS expression.

**Figure 1:**
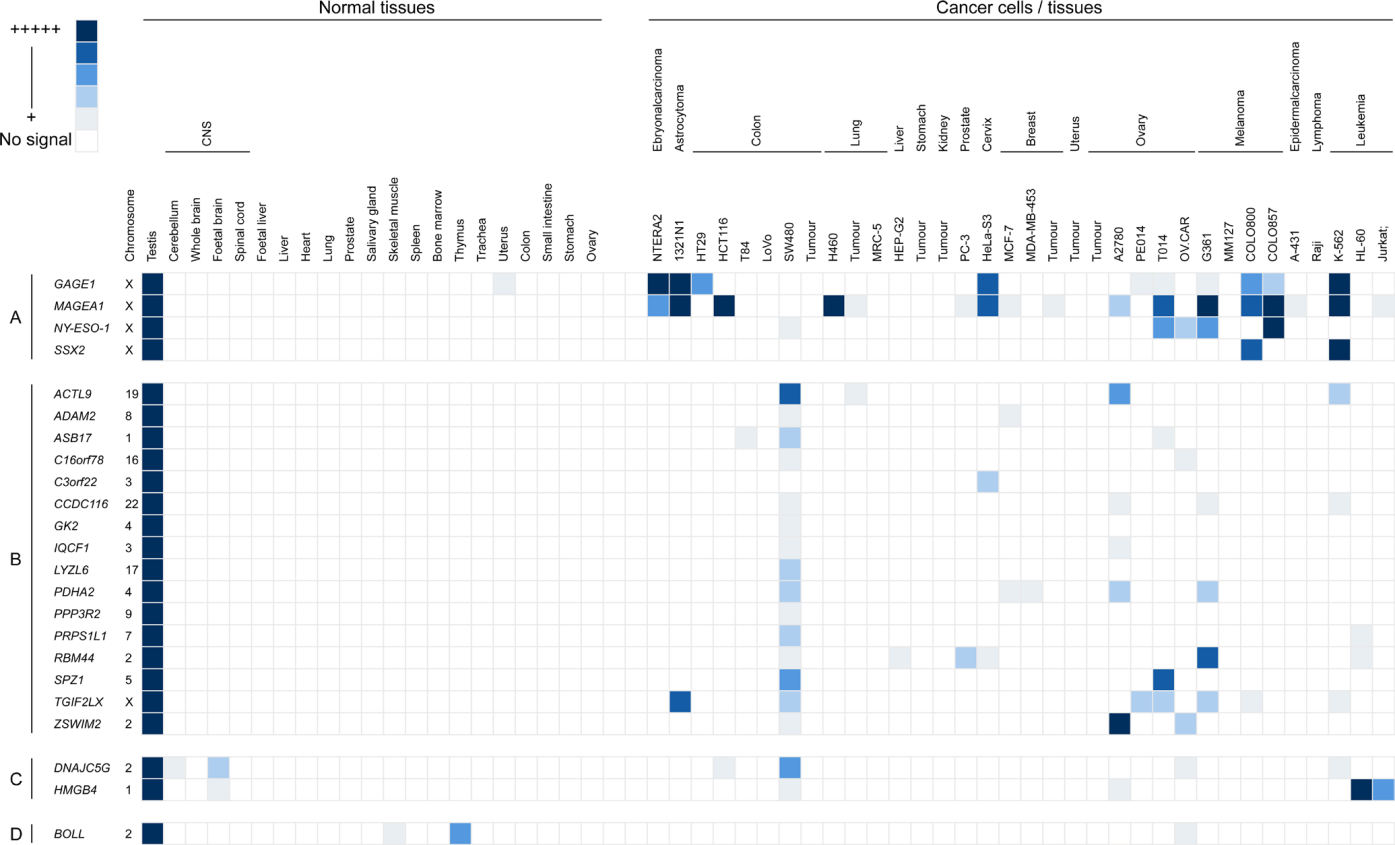
Grid representation of gene expression profiles for the 19 meiCT genes shown by RT-PCR to have expression in at least one cancer cell type Each gene has a lane allocation on the grid; the presence of a filled square in any column indicates that a positive RT-PCR signal was obtained (the shade of the filler indicates the intensity of the RT-PCR product on the agarose gel relative to the intensity of the band observed for the testis sample; the shade is not a reflection of the relative expression levels). Columns represent normal tissues (left hand set) and cancer cells/tissues (right hand set). Expression profiles for four previously characterised X chromosome encoded CTA genes have been shown as a positive control (set A: *GAGE1, MAGEA1, NY-ESO-1, SSX2*). The majority of the newly identified meiCT genes (16 genes; set B) had expression restricted to the testis in normal tissues, but exhibit expression in at least one cancer cell type. Two genes (*HMGB4* and *DNAJC5G*; set C) exhibited expression restricted to the testis and central nervous tissues in normal tissue samples. One gene (*BOLL*; set D) was expressed in testis, central nervous tissue and two other normal somatic tissues (thymus & skeletal muscle). The chromosomal location of the genes is given in the column to the right of the gene names.

The same 33 genes, which showed predominant (or only) expression in the testis (bold in Supplementary Table), were then analysed by RT-PCR in a range of cancer cell types. Following this analysis, 14 of these genes were shown to have no expression in any of the cancerous material (Supplementary Table). The expression profiles for those genes exhibiting cancer cell expression are shown in Figure 1. A further 16 genes were shown to have expression in at least one cancerous tissue and no expression in normal cells other than testicular cells (Figure 1, class B). Of the remaining 3 genes, 2 were cancer/testis/CNS restricted [i.e. expressed in at least one cancer cell type, in addition to the testis and normal CNS tissue (Figure 1, class C)] and 1 was cancer/testis-selective [i.e. expressed in one or two normal tissues other than CNS, as well as the testis and at least one cancer type (Figure 1, class D)].

### Meta-analysis of candidate genes expression profiles

**Figure 2:**
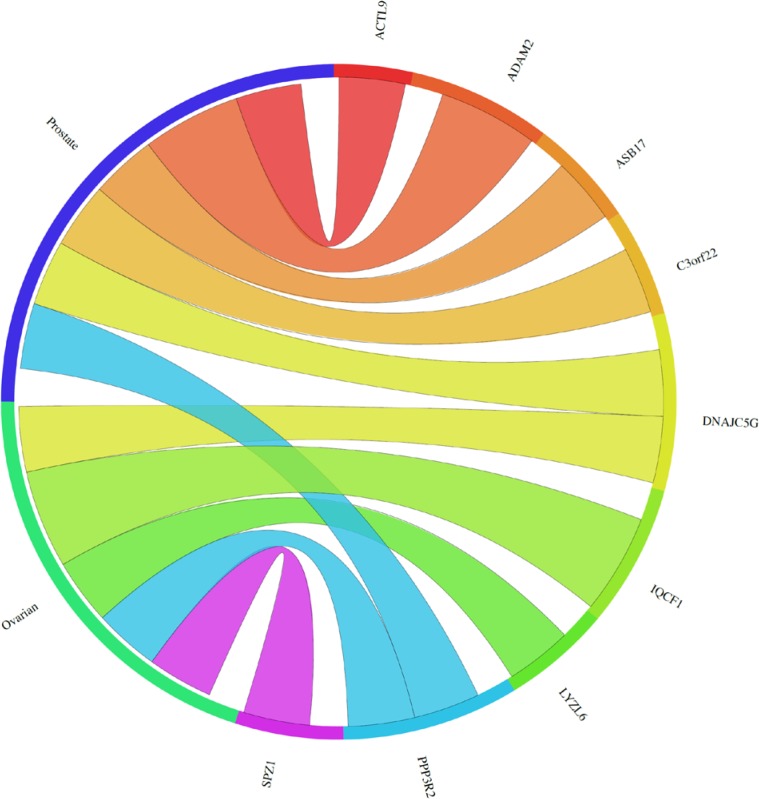
The Circos plot showing the meta-change in gene expression in relation to corresponding cancer types (ascribed by tissue type) for new meiCT genes that exhibited expression in at least one cancer cell type as assessed by RT-PCR (sets B-D in Figure 1; 18 of the 19 genes were subjected to the meta-analysis; TGIF2LX was not on the arrays). The plot shows 9 genes exhibit meta-up-regulation in ovarian and/or prostate cancers. The weight of the connection corresponds to the magnitude of the meta-change in gene expression.

**Figure 3:**
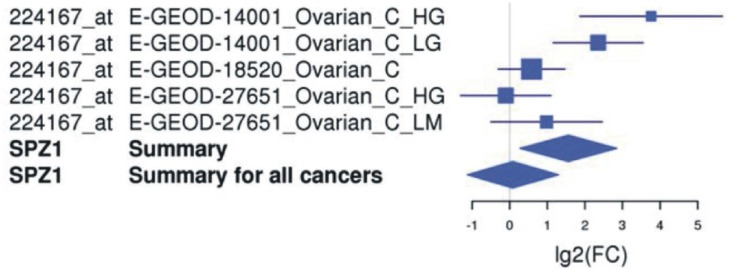
An example of a Forest plot for one of the newly identified meiCT genes, *SPZ1* *SPZ1* is up-regulated in ovarian cancers. The plot shows the 2-fold change for individual matched normal *vs*. tumour array sets [upper five horizontal lines; each study is illustrated by a square; the position on the x-axis represents the measure estimate (lg2-fold change ratio); the size is proportional to the weight of the study, and the horizontal narrow line reflects the confidence interval of the estimate], the total values for ovarian cancer (upper diamond; showing a significant up-regulation) and the total values for all cancers within the analyses (80 tumour *vs*. matched array sets for 13 cancer types [44]) showing no significant up-regulation for pooled values (lower diamond).

**Figure 4:**
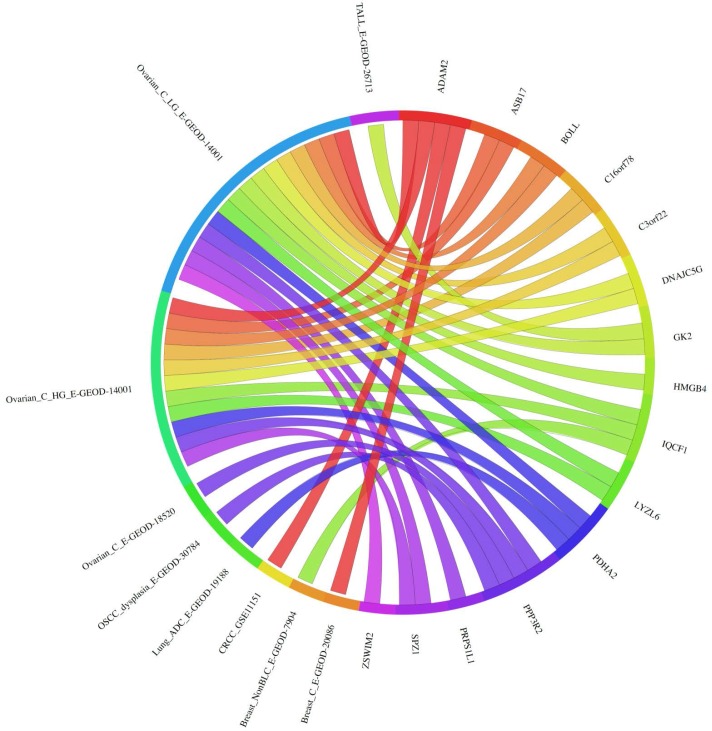
Circos plot for single microarray analysis for meiCT genes exhibiting expression in cancer cells/tissues as revealed by RT-PCR The expression of the genes is given corresponding to the data set in which a statistically significant up regulation was observed for the tumour *vs*. normal array analysis. For data set designations see [44] and [48]. The plot shows 15 of the 18 genes analysed are statistically significantly up-regulated in at least one cancer data set. 18 of the 19 genes showing cancer cell expression by RT-PCR analysis (Figure 1) were tested; three genes (*ACTL9, CCDC116, RBM44*) show no significant up-regulation in any of the array sets and so are not shown on the Circos plot (*TGIF2LX* was not on the arrays).

In order to explore the possible clinical relevance of the newly identified genes, we conducted meta-analyses using patient-derived cancer microarray data, including 13 cancer types in a range of 80 microarray data sets [48]. Firstly, we investigated the expression profiles of 18 of the 19 genes that exhibited expression in at least one of the cancer cell types tested by RT-PCR (1 was not present on the microarrays -*TGIF2LX*). Of these, 9 showed meta-up-regulation in either ovarian and/or prostate cancers (50.0%; Figure 2). An example of the meta-up-regulation of an individual gene (*SPZ1*) is given in the Forest plot profile for ovarian cancer in Figure 3. Whilst the meta-analysis reveals 9 genes to be up-regulated for two given cancer types (ovarian, prostate), analysis of single cancer data sets from the 80 cancer data sets used reveals evidence for activation of a total of 15 of the 18 candidate genes in at least one patient-derived sample set (83.3%; Figure 4).

**Figure 5:**
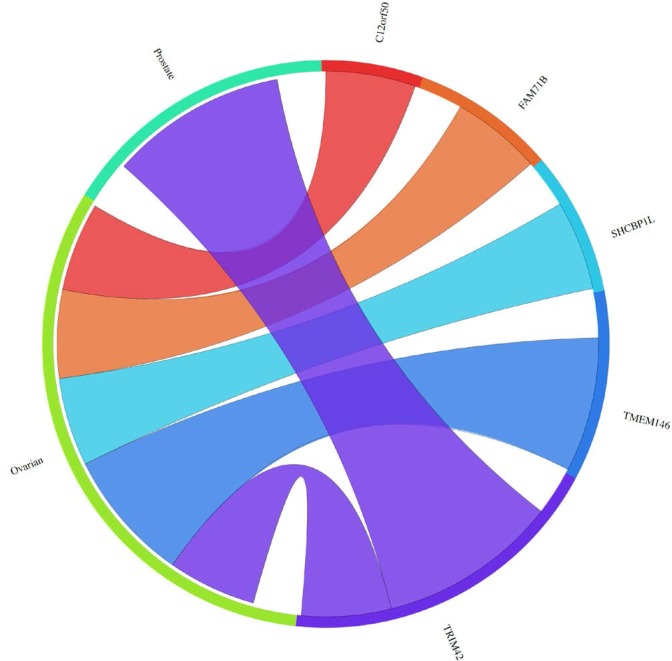
The Circos plot showing the meta-change in gene expression in relation to corresponding cancer types (ascribed by tissue type) for new meiCT genes that did not exhibit expression in at least one cancer cell type as assessed by RT-PCR [11 of the 14 genes (Supplementary Table) were subjected to the meta-analysis; 3 genes were not on the arrays (*C1orf141, HEATR7B, SATL1*)]. The plot shows 5 genes exhibit meta-up-regulation in ovarian and/or prostate cancers. The weight of the connection corresponds to the magnitude of the meta-change in gene expression.

**Figure 6:**
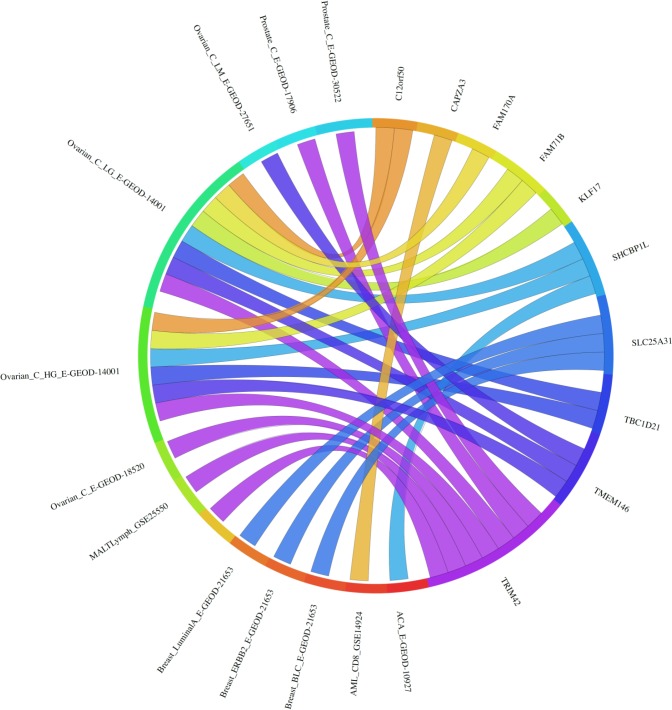
Circos plot for single microarray analysis for meiCT genes not exhibiting expression in cancer cells/tissues as revealed by RT-PCR The expression of the genes is given corresponding to the data set in which a statistically significant up-regulation was observed for the tumour *vs*. normal array analysis. For data set designations see [44] and [48]. The plot shows 10 of the 11 genes analysed are statistically significantly up-regulated in at least one cancer data set. 11 of the 14 genes showing no cancer cell expression by RT-PCR analysis (Supplementary Table) were tested (3 genes were not on the arrays - *C1orf141, HEATR7B, SATL1*); one gene (*C8orf74*) showed no significant up-regulation in any of the array sets and thus is not shown on the Circos plot.

Of the 33 genes with meiCT gene potential (based on expression patterns in normal tissue), 14 genes did not appear to be expressed in any of the cancer cell types analysed by RT-PCR (Supplementary Table). To further explore the possibility that these genes are meiCT genes, we used 11 of the 14 genes for meta-analyses using the 80 cancer gene expression microarray data sets (3 were not present on the microarrays, *C1orf141, HEATR7B* and *SATL1*) and found expression profiles for 5 of these genes were indicative of a cancer type marker for ovarian and prostate cancers (45.5%; Figure 5). A further 5 (10 genes in total) were expressed in at least one single cancer data set (Figure 6), indicating the potential to mark a specific sub-group of tumours within a cancer type. Only *C8orf74* exhibited no measurable expression in cancer cells / tissues (although *C1orf141, HEATR7B* and *SATL1* could not be analysed via meta-analyses due to their absence on the arrays).

## DISCUSSION

CTAs are cancer-specific biomarkers with considerable potential in prognostics/diagnostics and as therapeutic targets. The current classification system for CTA genes continues to be based on that put forward by Hoffman and colleagues [40]. We have since proposed a sub-category of CTA genes based on an *in silico* pipeline originating with putative meiotic genes; we have termed these meiCT genes [44]. Here we describe a further 29 genes which are novel meiCT genes. As for previously characterised meiCT genes, we found that most of these new genes are autosomally encoded (28 out of 29; Supplementary Table), a finding consistent with the transcriptional inactivation of the X chromosome during male meiosis [45,46]. An additional commonality with the previously characterised meiCT genes was the fact that many of this new cohort were shown to be up-regulated as a general marker for ovarian cancers: 10 of the new cohort displaying a meta-change increase in gene expression were in ovarian cancer. This again raises the possibility of using the meiCT genes to improve the diagnosis of this diverse and pernicious cancer type. It may be the case that genes that have a normal biological function (i.e. meiotic) in the foetal ovary are preferentially reactivated in cancers of this tissue type. Additionally, ovarian cancers are currently most frequently treated with cytoreductive surgery and chemotherapy although these types of tumours are immunoreactive and there is currently extensive work ongoing to explore the application of immune-based therapies for their treatment [49]. Thus, the identification of ovarian cancer-specific markers such as these is of exceptional potential therapeutic value [49].

Recent work has demonstrated that sub-groups of 26 germline and placental specific genes can be used to delineate aggressive metastasis-prone lung cancers [30]. This indicates that small sub-groups of tissue-specific genes can serve as accurate biomarkers in the stratification of complex and heterogeneous cancers. The clinical implications of this are far reaching as they offer extensive potential in establishing best practice approaches to therapeutic stratification. This work provides a paradigm for how germline gene expression in cancers can be applied to clinical stratification of complex disease. Having a definitive on/off expression profile, as observed with the meiCT genes, greatly enhances the potential simplicity of application of these genes in novel prognostics technologies.

A number of studies have now specifically explored the potential of expression of human germline genes as cancer biomarkers. Interestingly, whilst common genes have been identified however, the various studies have all identified additional distinct genes indicating that the full mining of data sets of this magnitude require multiple and diverse approaches. For example, this current study has identified 32 new genes with tight germline and germline/CNS tissue-specific expression restrictions (of the 33 genes analysed, *BOLL* was unique in exhibiting expression in two other somatic tissues, so was classed as testis selective); however, a recent seminal and extensive study of human male germline/placental genes only identified 21 of the 32 (65.6%) genes reported here as germline-specific [30]. A slightly lower trend is seen when analysing previously reported meiCT genes (22 out of 52 meiCT genes (42.3%) were reported as germline genes [30]; a total of 52.4% for all reported meiCT genes [44 and data presented here]).

In addition to serving as cancer biomarkers the meiCT genes may serve as therapeutic targets via a variety of routes. Firstly, the immunogenicity of the gene products of the meiCT genes remains very poorly characterised. Their highly stringent tissue specificity infers that their gene products could potentially serve as tumour-specific immunotherapeutic targets. Given the heterogeneity of cancer, both intra- and inter-tumour, the development of a large bank of markers/immunotherapeutic targets will be of increasing importance in personalisation strategies [5,50-52].

Lastly, germline genes in *D. melanogaster* serve to drive the oncogenic programme [38]. The human orthologues of these genes are also widely activated in human tumours [36] and other CTA genes have been demonstrated to be required for cancer cell proliferation and their depletion can serve to sensitise cancer cells to standard therapeutic agents (for example, see 33,34). Thus, not only can germline gene products potentially offer direct targets for drug therapies, but depletion of their activity can also serve to enhance the efficacy of existing therapies potentially enabling reduced dose regimes, which will limit undesired drug toxicities. The cancer-specific nature of meiCT gene expression makes these genes exceptionally attractive for further exploration in drug targeting and drug sensitisation.

The suggestion that germline genes are oncogenic infers that some of the genes identified here could play a tumour initiating / progression role. One of the genes validated here is a member of the *ADAM* gene family - *ADAM2*. The ADAM proteins often exhibit proteolytic activity and have emerging roles in the invasive properties of specialist cells within the placenta [53]. Such proteolytic functions may promote invasion and metastasis of solid tumours. A putative role for other members of the adamalysins in the aetiology and pathology of colorectal cancer and melanoma has been proposed [54,55]. This might indicate that not only are germline genes required for oncogenesis, they might drive metastasis and/or invasion and thus offer cancer-specific intervention points to stop the lethal spread of tumours.

*SPZ1* was shown to be testis-specific in our normal tissue panel, a finding previously shown by Hsu and colleagues [56]. In their study, they further showed that the gene was expressed both in the testis and epididymis. We found positive expression in a colon and ovarian cancer cell line and on meta-analysis there was a significant up-regulation in ovarian cancer. It has since been shown by Hsu and colleagues that *SPZ1*, which encodes a transcription factor, acts as a proto-oncogene to promote cellular proliferation and tumour formation in a mouse model [57]. Despite this, *SPZ1* is not a previously recognised CTA gene [18].

It has been suggested that another of the novel meiCT genes identified here, *SHCBP1L*, encodes a protein with strong homology to a mouse protein present in proliferating cells and may have similar physiological effects [58]. It remains unexplored whether this protein indeed acts through similar signal transduction pathways to promote proliferation but as the gene has shown a statistically significant meta-change up-regulation, again in ovarian cancer, makes this possibility worthy of further exploration.

### Closing remarks

Germline genes are emerging as important cancer-specific factors. The extent of their clinical importance is only now starting to come into focus, either as biomarkers for stratification and diagnosis, as oncogenic activators and as drug or immunotherapeutic targets. What is becoming increasingly apparent is the heterogeneity of tumour cell populations. This is the driver for the need to identify a large cohort of cancer cell markers that individually or in combination can target and mark a large number of tumour types and cell populations. Here we identify a new and extensive cohort of genes that can contribute to the growing catalogue of *bona fide* cancer-specific biomarkers.

## MATERIALS AND METHODS

### Cell lines and cell culture

The following cell lines were purchased from the European Collection of Cell Cultures (ECACC); 1321N1, COLO800, COLO857, G-361, HCT116, HT29, LoVo, MM127, SW480 and T84. The H460 cell line was purchased from the American Type Culture Collection (ATCC), and the two ovarian adenocarcinoma cell lines, PEO14 and TO14, were obtained from Cancer Research Techology Ltd. The NTERA-2 (clone D1) cell line was gifted by Prof. P.W. Andrews (University of Sheffield) and is regularly authenticated within the group using standard antibody tests using anti-OCT4 antibodies and retinoic acid-induced differentiation. The A2780 cell line was provided by Prof. P. Workman (Cancer Research UK Centre for Cancer Therapeutics, Surrey, UK) and was authenticated at source. Primary cultures of proliferating human prostate smooth muscle cells were obtained from PromoCell^TM^(C-12574). All cultures were used within a six months period of obtaining validated lines from external sources.

1321N1, A2780, NTERA-2 (clone D1) and SW480 cell lines were cultured in Dulbecco's modified Eagle's medium from Invitrogen^TM^ (DMEM + GLATAMAX^TM^) supplemented with 10% foetal bovine serum (FBS). COLO800, COLO857 and H460 cell lines were cultured in Invitrogens Roswell Park Memorial Institute 1640 medium (RPMI 1640) + GLUTAMAX^TM^ with 10% FBS. PEO14 and TO14 cell lines were cultured in RPMI 1640 + GLUTAMAX^TM^ supplemented with 10% FBS and 2mM sodium pyruvate, and MM127 was cultured in RPMI 1640 + GLUTAMAX^TM^ supplemented with 10% FBS and 25mM HEPES. McCoy's 5A medium + GLUTAMAX^TM^ supplemented with 10% FBS was used to culture the G-361, HCT116 and HT29 cell lines. Ham's F12 + DMEM (1:1) + GLUTAMAX^TM^ (Invitrogen^TM^) with 10% FBS was used to culture T84 cells.

All cell lines were grown in a 37°C incubator with 5% CO_2_, with the exception of the NTERA-2 (clone D1) cell line, which was grown at 37°C with 10% CO_2_.

### cDNA construction

Total RNA preparations were obtained from the human normal tissue panels (Clontech^TM^; 636643). RNA from tumour tissues and cell lines were purchased from Clontech^TM^ and Ambion^TM^. Total RNA was also isolated from cells using TRIzol (Invitrogen). Confluent cells were collected in TRIzol reagent and incubated at room temperature for 5 minutes. Chloroform was added with vigorous shaking and incubated for 5 minutes at room temperature. The aqueous phase was transferred to a clean tube following centrifugation at 12,000 *g* for 15 minutes at 4°C. The RNA was precipitated out of solution using isopropanol (10 minutes at room temperature and centrifuged at 12,000 *g* for 20 minutes at 4°C). RNA preparations were re-suspended in RNase-free water containing DNase. The concentration and quality of RNA was measured using a NanoDrop (ND_1000). 1.0 μg of total RNA was reverse-transcribed into cDNA using the SuperScript III First Strand synthesis kit (Invitrogen^TM^) as per the manufacturer's instructions.

### Reverse Transcriptase-Polymerase Chain Reaction (RT-PCR)

The sequences for each of the genes analysed were obtained from the National Center for Biotechnology (NCBI; http://www.ncbi.nlm.nih.gov/). Forward and reverse primers for each of the genes were designed, and where possible were intron-spanning.

A volume of 2 μl of diluted cDNA (containing ~150 ng/μl cDNA) was used for PCR in a 50 μl final volume. BioMix^TM^ Red and MyTaq^TM^ Red (Bioline^TM^) was used for PCR amplification. Samples were amplified with a pre-cycling hold at 96°C for 5 minutes, followed by 40 cycles of denaturing at 96°C for 30 seconds, annealing at a temperature between 54 and 60°C for 30 seconds and extensions at 72°C for 40 seconds followed by a final extension step at 72°C for 5 minutes. The products were separated on 1% agarose gels containing ethidium bromide or Gel Green^TM^.

## SUPPLEMENTARY TABLE


