# Supplementary material for: A novel cohort of cancer-testis biomarker genes revealed through meta-analysis of clinical data sets
Source: Oncoscience. 2014 May 6;1(5):349–59. doi: 10.18632/oncoscience.37 (PMC4278308; doi:10.18632/oncoscience.37)
Supplement: Supplementary file 1 [file oncoscience-01-0349-s001.pdf]

Supplementary Table. List of 54 potential class 4 meiCT genes.

Gene name	Ensembl ID	Unigene cluster ID	Chromosomal location
ACTL9	**ENSG00000181786**	**Hs.209206**	**19**
ADAM2	**ENSG00000104755**	**Hs.177959**	**8**
ASB17	**ENSG00000154007**	**Hs.125423**	**1**
BOLL	**ENSG00000152430**	**Hs 692026**	**2**
C1orf141*	**ENSG00000203963**	**Hs.666621**	**1**
C2orf61	ENSG00000239605	Hs.531575	2
C3orf22	**ENSG00000180697**	**Hs.178210**	**3**
C3orf30	ENSG00000163424	Hs.271580	3
C6orf81	ENSG00000157343	Hs.533066	6
C8orf74*	**ENSG00000171060**	**Hs.371776**	**8**
C9orf153	ENSG00000187753	Hs.632073	9
C10orf67	ENSG00000179133	Hs.522360	10
C12orf50 *	**ENSG00000165805**	**Hs.112930**	**12**
C16orf78	**ENSG00000166152**	**Hs.125875**	**16**
CAPZA3*	**ENSG00000177938**	**Hs.131288**	**12**
CCDC116	**ENSG00000161180**	**Hs.131615**	**22**
CCDC63	ENSG00000173093	Hs.437141	12
CCDC73	ENSG00000186714	Hs.706808	11
CCIN	ENSG00000185972	Hs.115460	9
DDI1	ENSG00000170967	Hs.591941	11
DNAJC5G	**ENSG00000163793**	**Hs.116303**	**2**
DYDC1	ENSG00000170788	Hs.407751	10
FAM170A*	**ENSG00000164334**	**Hs.713304**	**5**
FAM194A	ENSG00000163645	Hs.147128	3
FAM71B*	**ENSG00000170613**	**Hs.666099**	**5**
FIGLA	ENSG00000183733	Hs.407636	2
GK2	**ENSG00000196475**	**Hs.98008**	**4**
H2AFB1	ENSG00000198082	Hs.592246	X
HEATR7B2L*	**ENSG00000**	**Hs.97714**	**5**
HMGB4	**ENSG00000176256**	**Hs.568628**	**1**
IQCF1	**ENSG00000173389**	**Hs.671210**	**3**
KLF17*	**ENSG00000171872**	**Hs.567674**	**1**
LYZL6	**ENSG00000161572**	**Hs.97477**	**17**
PDHA2	**ENSG00000163114**	**Hs.131361**	**4**
PDILT	ENSG00000169340	Hs.376025	16
PPP3R2	**ENSG00000188386**	**Hs.151167**	**9**
PRPS1L1	**ENSG00000229937**	**Hs.169284:**	**7**
RBM44	**ENSG00000177483**	**Hs.720233**	**2**
RNF133	ENSG00000188050	Hs.126730	7
SATL1*	**ENSG00000184788**	**Hs.640783**	**X**
SHCBP1L*	**ENSG00000157060**	**Hs.497034**	**1**
SLC25A31*	**ENSG00000151475**	**Hs.149030**	**4**
SPDYA	ENSG00000163806	Hs.511956	2

SPZ1	**ENSG00000164299**	**Hs.519403**	**5**
TBC1D21*	**ENSG00000167139**	**Hs.124512**	**15**
TGIF2LX	**ENSG00000153779**	**Hs.592220**	**X**
TMEM146*	**ENSG00000174898**	**Hs.631842**	**17**
TRIM42*	**ENSG00000155890**	**Hs.343487**	**3**
TSGA13	ENSG00000213265	Hs.592266	7
TSSK2	ENSG00000206203	Hs.694070	22
UBL4B	ENSG00000186150	Hs.374027	1
UBQLN3	ENSG00000175520	Hs.189184	11
UMODL1	ENSG00000177398	Hs.242520	21
ZSWIM2	**ENSG00000163012**	**Hs.375054**	**2**

Note: embolden genes are those which were found to exhibited expression in two or fewer non-testis / central nervous system somatic tissues by RT-PCR on a range of normal human tissues.

*Signifies genes that exhibit not expression in cancer cells/tissues as assessed by RT-PCR validation using 34 cancer cell/tissue RNA sets.
